# Protoporphyrin IX iron(II) revisited. overview ofthe Mössbauer spectroscopic parameters of low spin porphyrin iron(II) complexes Part II

**DOI:** 10.1007/s00775-026-02158-9

**Published:** 2026-09-05

**Authors:** Jack Silver, Jehad A Taies, Husam M Abu-Soud, George R. Fern

**Affiliations:** 1 Department of Engineering, Wolfson Centre for Sustainable Materials Development and Processing, Brunel University of London, Kingston Lane, Uxbridge, UB8 3PH UK; 2 Department of Chemistry, College of Education for Pure Science, University of Anwar, Anwar, Iraq; 3 Department of Obstetrics and Gynecology, The C.S. Mott Center for Human Growth and Development, Wayne State University School of Medicine, 275 E. Hancock , Detroit, MI 48201 USA

**Keywords:** Iron Porphyrins, Mössbauer Spectroscopy, Myoglobin, Haemoglobin

## Abstract

Mössbauer parameters of low spin six coordinate [Fe(II)(Por)L_2_] and [Fe(II)(Por)LX] complexes (where Por is PPIX or a synthetic porphyrin; L is OH^−^, H_2_O, a nitrogenous base, CCl_2_, CO or CN^−^ and X is a different ligand to L) are discussed. Our point charge calculation approach is extended to investigate how the axial ligands and the four porphyrinato-N atoms generate the observed quadrupole splittings (ΔE_Q_). Partial quadrupole splitting (p.q.s.) and partial centre shift (p.c.s.) values are derived and discussed for all the axial ligands studied herein. It is shown that when the D_4h_ symmetry breaks down due to porphyrin ring ruffling, the point charge calculations no longer apply. This happens for many synthetic porphyrins but not for the uniquely important [Fe(II)(PPIX)] moiety, (which is ubiquitous in nature). Thus, synthetic porphyrins (like TPP) cannot always act as perfect proxies for natural haem proteins. In all the haem proteins containing [Fe(II)(PPIX)] moieties the haem is planar. Calculations indicate that if H_2_O were bound to the [Fe(II)(PPIX))] present in deoxyhaemoglobin or deoxy myoglobin, then these Fe(II) atoms would be low spin. In contrast for synthetic and natural haem proteins containing haem b (where the haem is connected to the protein backbone via two cysteine residues) the haem plane is ruffled (probably because of the methionine linkages transferring force and controlling the stereochemistry of the haem plane, and thereby its chemistry). For the [Fe(II)(TPP)(CN)_2_] and [Fe(II)(TPP)(1-MeIm)(CN)] the calculated CN^−^ ligand p.q.s values are suspect and probably show that the lattice charge is affecting them.

**Supplementary Information:**

The online version contains supplementary material available at https://doi.org/10.1007/s00775-026-02158-9.

## Introduction

Recently we reported an overview of the Mössbauer spectroscopic parameters of low spin porphyrin iron(II) complexes [1]. In that paper we showed how a well explored point charge calculation approach could be used to investigate how the L ligands and the four porphyrinato-N atoms in [Fe(II)(Por)L_2_] complex generate the observed Mössbauer quadrupole splitting (*ΔE*_*Q*_) values. Calculations were carried out to derive partial quadrupole splitting (p.q.s.) values and partial centre shift (p.c.s.) values for all the ligands we used and also for other similar ligands and Fe(II) porphyrins reported in the literature [1–4]. We were able to derive a large set of internally self-consistent p.q.s and p.c.s. values for all the low spin [Fe(II)(Por)L_2_] complexes in which the porphyrin was planar [1]. The p.c.s, and p.q.s. values for the ligands provided insight into the bonding into/towards the iron atoms and the effect on the porphyrin. In addition, the p.q.s. values for each porphyrin were found to be different and the results were used to emphasize the importance/uniqueness of the [Fe(PPIX)] moiety, which is ubiquitous in nature. The overall results have allowed a new understanding of the control that the porphyrin ligands exert on the low spin Fe(II) atom limiting its bonding to a defined range of axial ligands, outside of this range the spin state of the iron is not stable and intermediate spin or five coordinate high spin complexes are favoured.

In the same paper [1], we presented an analysis of the Mössbauer parameters, which enabled the construction of figures manifesting the relation between p.c.s, values and p.q.s. values. Evidence was found from some of the relationships presented in the figures that strong field ligands such as CO can and do change the sign of the field gradient in the [Fe(II)(Por)L_2_] complexes. Such figures can be used to forecast which ligands can cause this to occur with a particular porphyrin. The limiting p.q.s. value a ligand can have and still form a six-coordinate low spin [Fe(II)(Por)L_2_] complex was also established [1]. We found that if the two axial ligands had p.q. s. values that totalled less then around − 0.67 mm s^− 1^ then they were unable to generate a low spin [Fe(II)(Por)L_2_] complex. In fact, two axial ligands that had p.q.s. values close to this sum generated *ΔE*_*Q*_, of around 1.75 mm s^− 1^.

We did not extend our work [1] to consider the axial ligands H_2_O and OH^−^ as we did not have enough data to assign p.q.s. values to them. Herein, we present some new Mössbauer data and show how these ligands along with some contrasting strong binding ligands widen our understanding of the limits of the ΔE_Q_ values that [Fe(II)(Por)L_2_] complexes can have [5–9].

## Discussion

In Table 1 we present both new and relevant published Mössbauer data [5–7] and in Table 2 we present relevant crystal structural data of some of the complexes [7–28]. Our newly derived p.q.s. and p.c.s. values for the ligands studied in this work are presented in Table 3 along with some we previously derived [1] for comparison. To further characterise some of the complexes we also present their electronic spectral data in Table 4.

## Brief overview of the theory behind the derivation of p.q.s. and p.c.s. values

As we presented an in-depth background and derivation of p.q.s. and p.c.s. values we direct the reader to it and as all the relevant references are also given in that work [1], we will not reproduce them here. However, we summarise the equation for how the *ΔE*_*Q*_ can be calculated from the p.q.s. values and vice versa.

For six coordinate structures of tetradentate macrocyclic ligands all complexes are trans, so geometry is not in doubt but p.q.s. values are nevertheless still useful. They have been derived in full elsewhere [2] and so the reader is directed there. Briefly, they showed [2] assuming true octahedral angles and an asymmetry parameter of zero for tetragonally distorted trans FeA_4_B_2_ complexes that:

1 $$ DE_{Q} = + {\mathrm{4}}\left( {{\mathrm{p}}.{\mathrm{q}}.{\mathrm{s}}.} \right)_{{\mathrm{A}}} - {\mathrm{4}}\left( {{\mathrm{p}}.{\mathrm{q}}.{\mathrm{s}}.} \right)_{{\mathrm{B}}} $$

As a tetragonal distortion will result from both long or short axial bonds then an interesting consequence can arise; that if the axial bonds are long the sign of the field gradient will be positive but as they get shorter and stronger the sign can switch to be negative. By choosing an appropriate reference value of -0.30 mm s^− 1^ for coordinated chloride and using the known signs of the *ΔE*_*Q*_ parameters for a range of complexes, Bancroft et al. [2] assigned p.q.s. values to a large number of ligands. Dabrowiak et al. [3] extended these by including imidazole (Im) and R_2_NH where R = alky, aryl. We extended them still further in our previous works [1, 4].

As in our previous work [1] to calculate the p.c.s. values, starting values were taken from the work of Bancroft et al. [2] and our previous work [1] where appropriate and modified where necessary to best fit the data used in this work (Table 1 and refs 5–7). All Mössbauer chemical isomer shift **(**δ) data were corrected relative to stainless steel at 293 K where possible or 77 K so that the derived p.c.s. data can be readily compared to previously derived values.

As we wish to discuss the variation in bonding properties of the various ligands it is necessary to assume differences in δ are only due to differences in δ as did Bancroft et al. [2]

As

2 $$\delta = {\text{ }}\delta .{\text{ }} + {\text{ s}}.{\mathrm{o}}.{\mathrm{d}}. $$

Where second order Doppler shift (s.o.d.) includes the zero-point motion contribution. δ is dependent on the s-electron density at the nucleus and is thus sensitive to the strength of the chemical bonds formed by the Mössbauer atom. In contrast the s.o.d. is dependent on the structure of the solid and will vary from one solid to another [2]. Bancroft [2] noted that the temperature dependence of the δ of octahedral low spin iron(II) complexes are all similar [2] (0.07 ± 0.02 mm s^− 1^) suggesting that the temperature dependent part of the s.o.d. shift and any possible temperature dependence of δ are nearly constant [2]. Therefore, in the present calculations when only 77 K δ data are available, they are used by subtracting 0.07 mm s^− 1^ from the values. When the calculated value of δ is then shown for the compound (for comparison to 77 K data), 0.07 mm s^− 1^ is added to it.

Several p.c.s. values were taken from refs 1 and 2 (see Table 5 of ref 1) as a starting set and all other p.c.s. values were found by using these with the series of compounds in Table 1. We estimate that the errors on the p.c.s. values are less than ± 0.01 mm s^− 1^ as most of the complexes only have a δ error value of ± 0.01 mm s^− 1^. After Bancroft et al. [2] we assume agreement between observed and calculated data within ± 0.05 mm s^− 1^ is satisfactory and we have no results outside this range that we do not discuss in detail.

In our previous work [1], we discussed situations where the symmetry was no longer exactly D_4h_, yet it appeared that the model worked so long as the distortion from this symmetry was not too great. Here we will consider the limitations to this tetragonal model further. If ruffling of the porphyrin ring occurs, we will lose the four-fold axis, so, we go back to a C_2_, D_2_ or D_2h_ like symmetry. However, it is the electronic environment that is important and small symmetry distortions in the crystallographic environment may only have small effects on the electronic environment and not manifest as large changes in the electric field gradient and therefore not upset the correlations we will present herein. However, if an extreme change takes place, then the approach will not work. Previously [1] we did find evidence for extreme cases in some ruffled [Fe(II)(TMP)L_2_] complexes. In this work we will show evidence for similar extreme cases of ruffling and explain how this is relevant to some natural and modified proteins.

In the case of different axial ligands being present without ring-ruffling we get D_4_ symmetry since we lose the ring-symmetry plane. It appears this is acceptable if the electron density at the central Fe atom is not changing due to this ligand difference as appears to be true for the [Fe(II)(Por)(CO)L] where the calculations work well.

**Table 1:** ^57^Fe Mössbauer data for low spin [Fe(II)(Por)L_2_] and [Fe(II)(Por)LX] complexes

Compound	T ˚K	δ_obs_^*^	δ_calc_	ΔE_Qobs_	ΔE_Qcalc_	Ref.
		mm s^− 1^	mm s^− 1^	mm s^− 1^	mm s^− 1^	
[Fe(II)(TPP)(CCl_2_)(H_2_O)]	77	0.63(1)	0.63	1.11(1)	1.11	TW
[Fe(II)(TPP)(t-butNC)_2_]	77	0.40(1)	0.4	0.22(1)	0.22	TW
[Fe(II)(T4MepyP)(Im)_2_]^a^	77	0.54(2)	0.54	0.96(5)	0.96	TW
[Fe(II)(T4MepyP)(H_2_O)_2_]^b^	77	0.52(4)	0.52	1.75(7)	1.75	TW
[Fe(II)(T4MepyP)(CO)(H_2_O)]^c^	77	0.38(5)	0.38	0.79(3)	0.80.	TW
[Fe(II)(PPIX)(CO)(OH)]^−d^	77	0.37(2)	0.35	0.92(2)	0.94	TW
[Fe(II)(PPIX)(CO)(OH)]^−e^	77	0.34(2)	0.35	0.94(2)	0.94	TW
[Fe(II)(TPPS)(-C_6_H_4_SO_3_^−^)_2_]^f^	78	0.55(2)	0.55	0.97(2)	1.09	5
[Fe(II)(TPPS)(-C_6_H_4_SO_3_^−^)_2_]^g^	78	0.55(2)	0.55	1.20(2)	1.09	5
[Fe(II)(TPPS)(-C_6_H_4_SO_3_^−^)_2_ ]^h^	78	0.53(2)	0.55	1.12(2)	1.09	5
[Fe(II)(TNPS)(-C_6_H_4_SO_3_^−^)_2_]^i^	78	0.47(1)	0.51	1.09(1)	1.13	6
[Fe(II)(TNPS)(-C_6_H_4_SO_3_^−^)_2_]^j^	78	0.52(1)	0.51	1.22(1)	1.13	6
[Fe(II)(TNPS) )(-C_6_H_4_SO_3_^−^)_2_]^k^	78	0.54(1)	0.51	1.10(2)	1.13	6
[Fe(II)(TNPS)(CO)(H_2_O)]	78	0.37(1)	0.37	0.72(2)	0.79	TW
[Fe(II)((SPh)_2_(SMePh)_2_P)(-C_6_H_4_SO_3_^−^)(H_2_O)]^l^	78	0.62(2)^m^	0.635	1.49(3)	1.45	TW
	78	0.65(2)^n^	0.635	1.41(1)	1.45	TW
[Fe(II)((SPh)_2_(SMePh)_2_P)(-C_6_H_4_SO_3_^−^)_2_]^o^	78	0.60(1)	0.6	1.21(1)	1.21	TW
[Fe(II)(TPBP)(t-butNC)_2_]	100	0.394(4)	0.394	0.197(5)	0.2	7

^8^ All δ values are relative to stainless steel, ^a^ frozen solution, compound included to facilitate the calculation of a p.q.s. value for T_4_MepyP; ^b^ frozen solution at pH = 2.6; ^c^ frozen solution at pH = 9.0; ^d^ frozen solution of DMF-H_2_O; ^e^ frozen solution pH = 9.0; ^f^ frozen solution at pH = 6.7; ^g^ frozen solution at pH = 10.0; ^h^ frozen solution at pH = 11.2; ^i^ frozen solution at pH = 10.0; ^j^ frozen solution at pH = 11.6; ^k^ frozen solution at pH = 14; ^l^ Species is dominant over the range pH 6.3 to 14.0 ; ^m^ at pH 6.3; ^n^ at pH 9–14; ^o^ Frozen solution at pH = 11.5 more concentrated solution, 250 mg of the [Fe(III)((SPh)_2_(SMePh)_2_P)] solid in 2 ml of water with a few drops of sodium dithionite added to the solution

**Table 2:** Crystal Structure data, ^57^Fe Mössbauer data and p.q.s. values for low spin [Fe(Por)L_2_)] complexes, [Fe(II)(Por)(X)(Y) complexes and [Fe(Por)L(CO)] complexes

Compound	*N*_ax_-Fe Å	L_ax_-Fe Å	*N*_Por_-Fe Å	ΔE_Qobs_	*p*.q.s.	Refs.
		(where L = other axial ligand(s))		mm s^− 1^	mm ^s−1^	
[Fe(TPP)(pip)_2_]	2.127(3)		2.004(6)	1.52	-0.405	10, 11
[Fe(TPP)(py)_2_]	2.037(1)		2.001(2)	1.22	-0.46	12,13,14, 15
[Fe(TPP)(1-VinylIm)_2_]	2.004(2)		2.001(2)	1.02	-0.5	16
[Fe(TPP)(1-BzylIm)_2_]	2.017(4)		1.993(9)	1.02	-0.5	16
[Fe(TPP)(1-MeIm)_2_]	2.014(5)		1.997(6)	1.07	-0.5	16
[Fe(TPP)(CCl_2_)(H_2_O)]		Fe-C = 1.83	1.984(4)	1.11(1)		8, TW
		Fe-O = 2.13				
[Fe(TPP)(py)(CO)]	2.10(1)	Fe-C = 1.77(2)	2.02(3)	0.57	-0.46	13, 17
[Fe(TPP)(1-MeIm)(CO)]	2.071(2)	Fe-C = 1.793(3)	2.003(5)	0.35	-0.5	13, 18
[Fe(TPP)(t-butNC)_2_]		Fe-C = 1.901 (3)	2.005(4)	0.22		9, TW
[Fe(TPBP)(t-butNC)_2_]		Fe-C = 1.907(2)	2.007(17)			7
[Fe(TFPP)(Fe(C_5_H_5_)(C_4_H_4_N))_2_]	2.05(2)		2.01(2)	1.25		19
[Fe(TpivPP)(1-MeIm)_2_]	1.9958(19)		1.992(3)	1.05	-0.5	20,
[Fe(TpivPP)(1-EtIm)_2_]	2.0244(18)		1.993(6)	1.07	-0.5	20,
[Fe(TpivPP)(1-VinylIm)_2_]	1.9979(19)		1.988(5)	1.07	-0.5	20,
[Fe(Tpiv_2_ C_12_P)(1-MeIm)(CO)]	2.062(5)	Fe-C = 1.728(6)	1.999(3)	0.27	-0.5	21, 22, 23
[Fe(II)(OEP)(1-MeIm)(CO)]	2.077(3)	Fe-C = 1.744(5)	2.000(3)	0.4	-0.5	24, 25
[Fe(II)(TPP)(1-MeIm)(CO)]^a^	2.0503(14)	Fe-C = 1.7600(17)	2.005(6)	0.32	-0.5	24,(26)
[Fe(TMP)(4-CNpy)_2_]	1.996(2)		1.993(2)	1.13	-0.47	26,
[Fe(TMP)(4-Mepy)_2_]	2.010(2)		1.988(2)	1.12	-0.47	26,
[Fe(TMP)(2-MeHIm)_2_]	2.047(3)2.030(3)		1.964(5)	1.78	0.3475	27,
[Fe(TMP)(2-MeHIm)_2_]	2.032(3)2.028(3)		1.961(7)	1.78	0.3475	27,
[Fe(PMXPP)(amp)_2_]	2.037(2)		1.988(2)	1.04	-0.52	28

^a^ This structure is slightly different to the one given higher in this table

We first present how we calculated the p.q.s. values for the porphyrin ligands and axial ligands present in the compounds (see Table 3). In some cases, we previously wrongly assigned the identity of the axial ligands in frozen solutions of the Fe(II) porphyrin complexes [5, 6], the current analysis has allowed us to reconsider some of our previous suggestions, and so we take this opportunity to reassign the axial ligand identities.

**Fig. 1:**
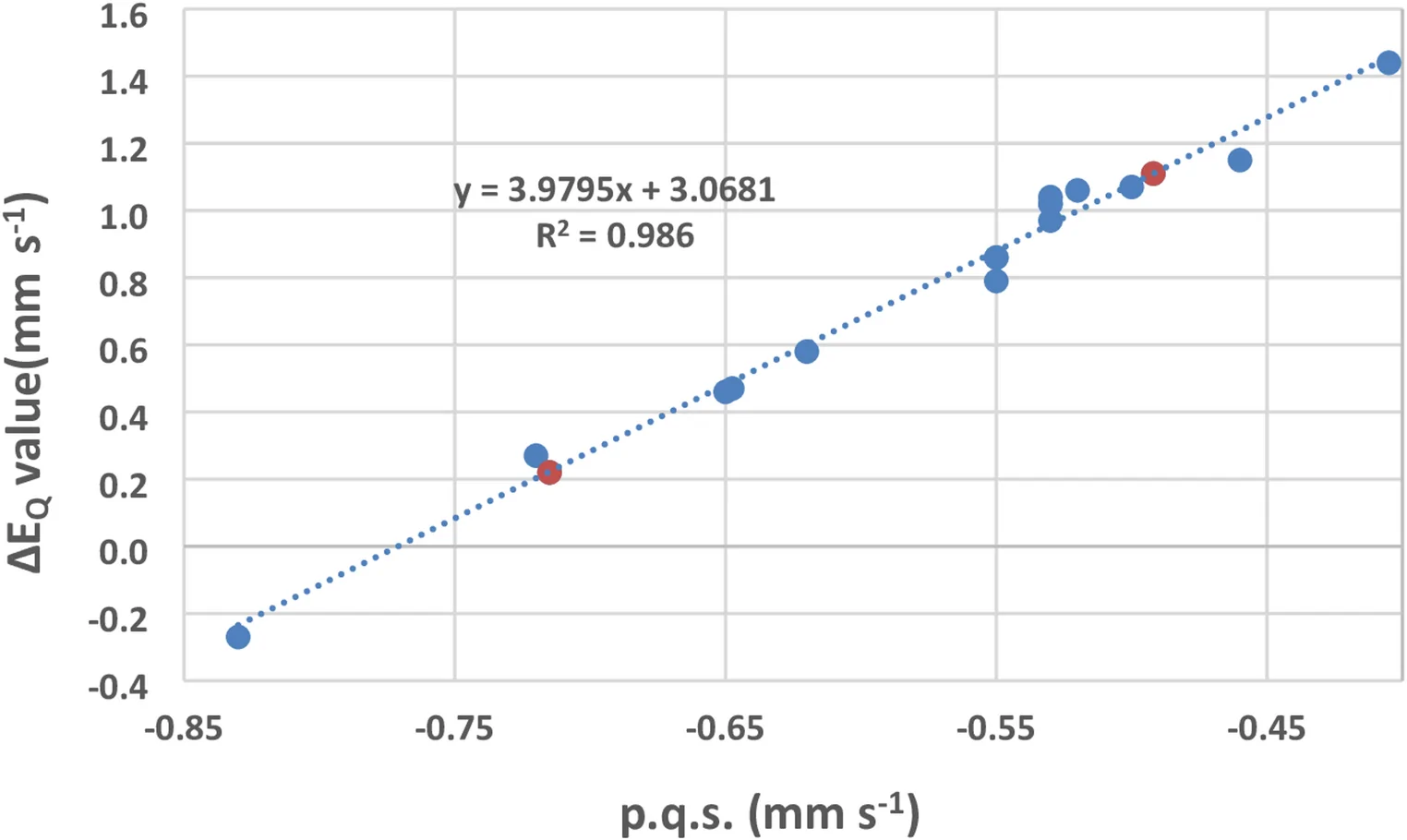
Plot of ΔE_Q_ value against the axial ligand p.q.s. value for [Fe(II)(TPP)L_2_] complexes. The red circles are the two complexes presented in this work. The other 16 blue circles were discussed previously [1]

Low spin [Fe(II)(TTP)(*t*-butNC)_2_] [9] has two axial isocyanide ligands at a distance of 1.901(3) Å from the Fe atom (see Table 2). It has a very small *ΔE*_Q_ (0.22 mm s^− 1^) that can be used to calculate its p.q.s. value to be -0.715 mm s^− 1^. This fits on the linear regression line (see Fig. 1) we previously derived for the [Fe(II)(TPP)L_2_] complexes [1], and from our previous work we can say that the sign of the field gradient in this complex is positive. It is not surprising that the related complex [Fe(TPBP)(t-butNC)_2_] [7] complex has a similar small *ΔE*_*Q*_ and very similar bond lengths (see Table 2). Unfortunately, there are not enough low spin [Fe(II)(TPBP)L_2_] complexes to define the p.q.s. value of this porphyrin accurately (but it is probably a little larger than that of TPP). It is worth noting that Bancroft et. al. [29] derived a p.q.s. value of -0.69 mm s^− 1^ for *iso*-cyanide ligands (RNC) in non-porphyrin containing complexes which is close to our value for t-butNC (see Table 3).

**Table 3:** P.c.s and p.q.s. for ligands bound to octahedral low-spin iron(II)^a^ in [Fe(Por)L_2_] complexes

Monodentate Ligands	*p*.c.s. (mm s^− 1^)	*p*.q.s. (mm s^− 1^)	Macrocyclic Ligand	*p*.c.s. (mm s^− 1^)	*p*.q.s. (mm s^− 1^)
Im	0.08^b^ (0.10)^c^	(0.46)^c^ -0.52^d^			
Pip	0.08^b^	-0.405^d^	0.25Pc	0.05^d^	-0.965^d^
Py	0.07^d^ (0.07)^b^	-0.46^d^	0.25PMEPP	0.0875^d^	-0.79^d, e^
NH_3_	0.07^d^	-0.52^d^	0.25PMXPP	0.0825^d^	-0.78^d, e^
n-but	0.07^d^	-0.49^d^	0.25PClPP	0.0825^d^	-0.775^d, e^
s-but	0.07^d^	-0.5025^d^	0.25TMP	0.875^d^	-0.7725^d^
t-but	0.10^d^	-0.4875^d^	0.25TpivPP	0.9^d^	-0.77^d^
His	0.05^d^	-0.50^d^			
			0.25TNP	0.075^d^	-0.765^d^
			0.25OEP	0.0775^d^	-0.765^d^
t-butNC	0.02	-0.715	0.25TPP	0.09^d^	-0.77^d^
H_2_O	0.07	-0.3225	0.25PPIX	0.09^d^	-0.76^d^
OH^−^	0.01	-0.24	0.25T4MepyP	0.0775	-0.76
CO(PPIX)	0.0	-0.81^d^	0.25TPBP	0.071	-0.7625
CO(T4MepyP)	0.0	-0.80	0.25T(SPh)_2_(SMePh)_2_P	0.0775^d^	-0.765^d^
CCl_2_	0.13	-0.6625	0.25TNPS	0.075^d^	-0.765^d^
			0.25TPPS	0.08^d^	-0.7675^d^
SO_s_^−^ of TPPS	0.08	-0.496			
SO_3_^−^ of TNPS	0.07	-0.48			
			0.25T4MepyP with CO and H_2_O	0.06	-0.76
CO(PPIX) with OH^−^	0.0	-0.81	0.25PPIX with CO and OH^−^	0.0675	-0.76
CO(T4MepyP) with H_2_O	0.0	-0.851	0.25TNPS with CO and H_2_O	0.574	-0.765
CO(TNPS) with -C_6_H_4_SO_3_^−^	0.0	-0.847			
CN(TPP)	0.02^d^	-0.73^d^			
CN(PPIX)	0.02^d^	-0.76^d^			
CN(TPPS)	0.02^d^	-0.77^d^			
CS	-0.12^d^	-0.80^d^			

^a^ All values calculated from δ relative to stainless steel at 298 K

^b^ Value according to Bancroft et al., (refs 2, 29)

^c^ Value according to Dabrowiak et al., (ref. 3). Value in parenthesis not used in this work

^d^ Value according to Silver et al.,(ref 1)

^e^ Calculated from room temperature data;

To establish p.q.s. values for the two axial ligands in the complex [Fe(II)(TPP)(CCl_2_)(H_2_O)] it is first necessary to calculate a p.q.s. value for H_2_O. This can be achieved from the data presented in Table 1. A p.q.s. value of -3.04 mm s^− 1^ for the porphyrin T4MepyP can be found from [Fe(II)(T4MepyP)(Im)_2_] using a p.q.s. value of -0.52 mm s^− 1^ for the Im ligand [1]. Then from the complex [Fe(II)(T4MepyP)(H_2_O)_2_] a p.q.s. value of -0.3225 mm s^− 1^ can be calculated for H_2_O. The p.q.s. value for CCl_2_ is then − 3.08 - -1.11 = -1.97–2 x -0.3225 = -1.325/2= -0.6625 mm s^− 1^. By taking the average value of the two p.q.s. values for the two axial ligands in the complex [Fe(II)(TPP)(CCl_2_)(H_2_O)] and plotting that against the *ΔE*_*Q*_ value for the complex this also fits the linear regression line in Fig. 1, and the correlation is still very good (R^2^ = 0.986). Thus the p.q.s. value for H_2_O is good. However, we note that Bancroft et al. [29] derived a p.q.s. value of -0.44 mm s^− 1^ for H_2_O which is not within experimental error of our value. It should be noted that if we used the p.q.s. value for H_2_O from Bancroft [29] then the calculated *ΔE*_*Q*_ value (1.28 mm s^− 1^) for [(Fe(II)(T4MepyP)(H_2_O)_2_] would be very far out from the experimental value (1.75 mm s^− 1^). It appears that our value is more reasonable. It is worth restating that it is important to have a self-consistent set of p.q.s. values as we previously derived [1] using a large data set, in fact we never relied on less than three *ΔE*_*Q*_ values to derive a p.q.s. value. We note in the case of Bancroft’s value [29] for H_2_O he had only one *ΔE*_*Q*_ value, moreover this was based on K_3_[Fe(II)(CN)_5_(H_2_O)].7H_2_O in which the octahedral structure may well have been distorted, we note that others [30] reporting p.q.s. values for complexes of the type Na_3_[Fe(II)(CN)_5_(X)]nH_2_O (where X is a nitrogenous ligand) list values that vary inconsistently from ours. In contrast another group [31] who derived p.q.s. values for the same ligands in [Fe(II)(Pc)(X)_2_] complexes agree well with our values. It thus appears that for planar macrocyclic ligands’ self-consistent p.q.s. values for axial ligands and the macrocyclic ligands agree well between different groups of workers.

The compound [Fe(II)(T4MepyP)(H_2_O)_2_] had a *ΔE*_Q_ value of 1.75 mm s^− 1^; this value is close to the maximum value that we previously identified as the largest possible for a low spin [Fe(II)(Por)L_2_] complex [1]. Thus, the p.q.s. value of -0.3225 mm s^− 1^ for H_2_O is close to the smallest found that can stabilize a low spin [Fe(II)(Por)L_2_] complex. Moreover, [Fe(II)(T4MepyP)(H_2_O)_2_] is only stable at very low pH (2.6) and as the pH increase only high spin Fe(II) species are present in the frozen solutions. Indicating that a positively charged [Fe(II)porphyrin] complex will attract OH^−^ ions when the concentration in solution allows it and this leads to the formation of the high spin [Fe(II)(T4MepyP)(OH)]^−^.

**Fig. 2:**
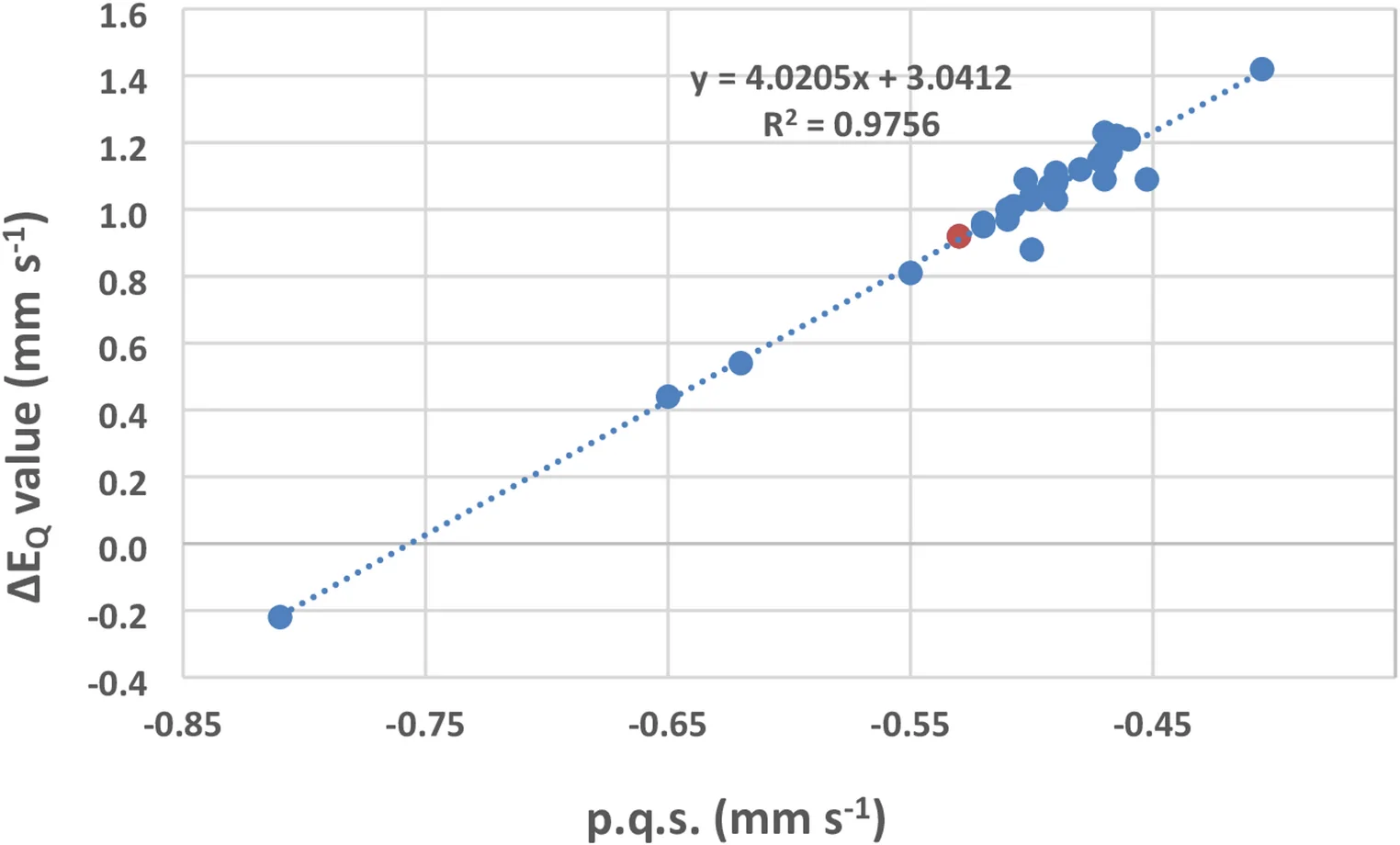
Plot of ΔE_Q_ value against the axial ligand p.q.s. value for [Fe(II)(PPIX)L_2_] complexes. The red circle is the [Fe(II)(PPIX)(CO)(OH)^−^] complex discussed in this work, the other 36 complexes were discussed previously

The next objective was to identify the nature of the sixth ligand in the frozen solutions containing [Fe(II)(PPIX)(CO)X] (where X = H_2_O or OH^− 1^ ) complexes. When Por = PPIX then the p.q.s. for CO = -0.81 mm s^− 1^ [1] and the p.q.s value for the sixth ligand is -3.04 - -0.92- -1.62 = -0.5/2 = -0.25 mm s^− 1^. Thus, X in [Fe(II)(PPIX)(CO)X] is clearly not a H_2_O molecule but more likely to be a OH^−^. However, the complex labelled [Fe(II)(T4MepyP)(CO)(H_2_O)]^−^ would generate a p.q.s. value of 0.31 which is close to the p.q.s. value for H_2_O. It would thus appear that the two CO complexes stabilize different axial ligands, this is surprising as the two porphyrins have the same p.q.s. values, however they have different substituents which means they will react differently with the surrounding solutions. We have plotted the average p.q.s. value of the axial ligands for the [Fe(II)(PPIX)(CO)(OH)^−^] complex against its *ΔE*_*Q*_ value in Fig. 2, the resulting linear regression line correlation is also still good, so the p.q.s. value for OH^−^ seems very reasonable. We have previously shown that CO can stabilise a weak axial ligand in a six coordinate low spin [Fe(II)(Por)(CO)(L)] complex, in that case L was a sulfur coordinating ligand [1].

**Fig. 3:**
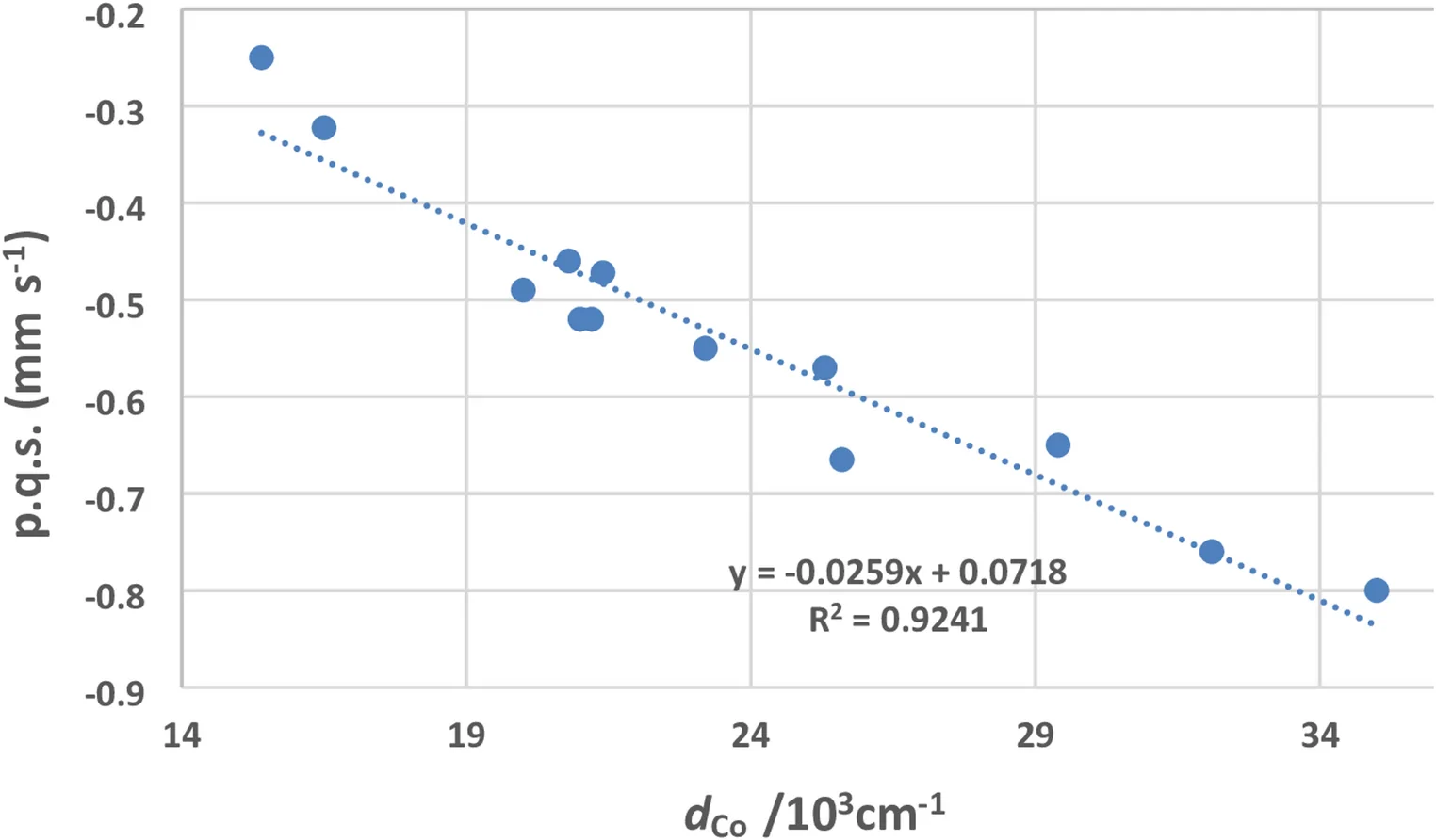
Plot of *d*_Co_/10^3^cm^− 1^ (where *d*_Co_ is the *d* value of each ligand for different complexes of a given metal and can be evaluated as in ref. 32 from where the *d*_Co_ values are taken). *d*_Co_ can be correlated to Jorgenson’s *f* (ligand) value [33] against the p.q.s. values of eleven axial ligands (derived in our earlier work [1] and two in this work

We have shown [1] that the *d* parameter (proposed for the spectrochemical series of ligands in the mixed ligand complexes of d^6^ metals that allowed the prediction of low energy d-d absorption bands in a d^6^ complex that was proposed by Shimura [32]) can be plotted against the p.q.s. values of the axial ligands giving a very reasonable correlation. Shimura [32] deduced a series of *d* (in 10^3^ cm^− 1^) values for over 100 monodentate ligands and many bidentate ligands for Co(III) complexes and explained how they could be adapted for any d^6^ metal. We have extended our previous plot [1] and added the values for H_2_O and OH^−^, the correlation is still very reasonable (see Fig. 3) and there are now thirteen ligands in the plot.

We previously published the results of studies on [Fe(II)(TPPS)] [5] and [Fe(II)(TNPS)] [6] in frozen aqueous solution as a function of pH. Both series of complexes showed evidence for low spin [Fe(II)(Por)(L)(L’)] complexes at lower pH values. We suggested that these compounds were likely to have axial bonds to water molecules. In the light of our current knowledge (discussed above) this cannot be correct, so we now search for a more reasonable explanation. The *ΔE*_*Q*_ values of these low spin Fe(II) complexes were small in the range of 0.97 mm s^− 1^ to 1.22 mm s^− 1^ (see Table 1) suggesting the ligands must have relatively large p.q.s. values. From the discussion above the ligands in these complexes are not H_2_O or OH^−^. The most likely axial ligands for these [Fe(II)(Por)(L)(L’)] are the SO_3_^−^ type ligands that are on the porphyrin macrocycles or the SO_x_^−^ ligands that remain from the dithionite reduction used to generate [Fe(II)(T4Mepy)] species. At the end of this paragraph, and in the following paragraph we discuss evidence to rule out the possibility of the ligands being due to the dithionite, and thus they must be from the surrounding porphyrin molecules. Clearly the low spin [Fe(II)(TPPS)(L)(L’)] and [Fe(II)(TNPS)(L)(L’)] complexes seem to change as a function of pH (see values in Table 1). In the pH range 5.0 to 11.2 the electronic absorption spectra (see Table 4) do not change at room temperature for the low spin [Fe(II)(TPPS)(L)(L’)] complex suggesting only one species is present; so, in frozen solution the changes observed in the *ΔE*_*Q*_ values of the complex are likely to be due to H-bonding interactions between the axial ligands and the water molecules. In the case of the low-spin [Fe(II)(TNPS)(L)(L’)] complex in solution in the pH range 7.2 to 12.1 there is evidence for only one species from the electronic absorption spectra (see Table 4), and this is different to the species in the [Fe(II)(TPPS)(L)(L’)], however at lower concentration the spectra are very similar to the later species with all the bands red shifted by 4–6 nm (we previously suggested the shift was due to the change of a naphthyl group for a phenyl group) [6], Again in frozen solution the changes we observe in the *ΔE*_*Q*_ values with pH of the low spin species are likely to be a consequence of H bonding to the axial ligands present on the Fe(II) centre. It is worth noting that in similar studies on [Fe(II)(PPIX)] [34, 35] and [Fe(II)(TCPP)] [36] frozen solutions we saw no evidence for low spin Fe(II) complexes as a function of pH.

If the remaining dithionite ions in the reduced iron porphyrin frozen solutions could act as axial ligands, then we might expect all frozen solutions that contain dithionite reduced iron porphyrins to bind to dithionite ligands and thus give low spin complexes with ΔE_Q_ values in the same range. Clearly there is no evidence for this, in the case of Fe(II)(PPIX) frozen solutions there is no evidence for a low spin complex [34, 35]; the same is true for Fe(II)(TCCP) complexes [36]. In addition, the low spin complex in the Fe(II)(T_4_MepyP) frozen solutions has a very much larger ΔE_Q_ value than those of the Fe(II)(TPPS) and Fe(II)(TNPS) frozen solutions so clearly the axial ligands are different. This evidence rules out bonding to dithionite as a ligand in low spin Fe(II)(Por) complexes as it is present in all the Fe(II)(Por) frozen solutions and the low spin complexes are only present in three cases. The evidence for these conclusions is seen in the Mossbauer spectra in frozen solution and in the electronic spectra [5, 6, 34–36].

**Table 4:** Electronic absorption spectra of low spin [Fe(II)(Por)(L)(L’)] solutions

Compound	pH	Soret				Refs.
		λ (nm)	λ_2_ (nm)	λ_3_ (nm)	λ_4_ (nm)	
[Fe(II)(TPP)(CCl_2_)(H_2_O )]^a^		409	527	551		TW,
		408	525	550		8
[Fe(II)(TPP)(t-butNC)_2_]^b^		431 (412sh)	539	573		TW,
		432 (411sh)	537	572		9
[Fe(II)(TPBP)(t-butNC)_2_]^c^		437 (413sh)	543	579		7
[Fe(II)(T4MepyP)(H_2_O)_2_]^d^	2.5	429	543		628	TW
[Fe(II)(PPIX)(CO)(OH)]^− d^	9	410	525	565		TW
[Fe(II)(TPPS)(L)(L’)]^d^	7	424		555	596	5
[Fe(II)(TPPS)(L)(L’)]^d^	10.2	424		557	598	5
[Fe(II)(TPPS)(L)(L’)]^d^	11.2	424		557	598	5
[Fe(II)(TNPS)(L)(L’)]^de^	8.6	431	537		602	6
[Fe(II)(TNPS)(L)(L’)]^de^	11.3	432	535	564	603	6
[Fe(II)(TNPS)(L)(L’)]^de^	14	444		570	608	6
[Fe(II)(SPh)_2_(SMePh)_2_P)(L)(L’)]^f^	6.7	424	538	555	596 (sh)	TW
[Fe(II)(SPh)_2_(SMePh)_2_P)(L)(L’)]^f^	11.4	424	537	564	598 (sh)	TW

^a^ chloroform/methanol, some [Fe(II)(TPP)(CCl_2_)] was also present; ^b^ in toluene; sh=shoulder; ^c^ in chloroform; ^d^ aqueous solution; ^e^ concentration of solutions varied to facilitate obtaining features of the spectrum (see ref 6 for detailed discussion); ^f^ concentrations were varied to facilitate obtaining features of the spectrum. At pH 11.4 and above there was evidence for a high spin Fe(II) species in the Mössbauer spectra in frozen solution

In the case of the [Fe(II)(T4MepyP)] solution the low spin [Fe(II)(T4MepyP)(H_2_O)_2_] complex only exists at very low pH 2.6 and its electronic absorption spectra (see Table 4) is different to those referred to above. As stated above, all these solutions contained dithionite as a reducing agent, it is unlikely to be the axial ligand causing the low spin [Ee(II)(TPPS)(L)(L’)] and [Fe(II)(TNPS)(L)(L’)] complexes so the most likely candidate are the -SO_3_^−^ groups on the porphyrins of neighbouring molecules. If we assume that the -SO_3_^−^ are the axial ligands, then we can derive a p.q.s. of around − 0.49 mm s^− 1^ for them. This value is close to those we reported for alkyl amines and imidazole. Thus, it appears that the -SO_3_^−^ groups act as strong bonding ligands to the Fe(II) atom in these sulfonated iron porphyrins. There is a crystal structure of a [Mo(O)(TMP)(C_6_H_5_SO_3_)] [37] in which the -SO_3_^−^ is bonded to the Mo atom via one of its O atoms (this Mo-O bond length is 2.179(7) Å). The Mo atom is in the porphyrin plane and thus this crystal structure is a good model for the proposed bonding of the -SO_3_^−^ groups to the Fe(II) atoms discussed above. The rest of the -C_6_H_4_SO_3_ molecule sits above the porphyrin plane sloping upwards gradually from the oxygen. It is thus possible that in the [Fe(II)(TPPS)(L)(L’)] complexes that other [Fe(II)(TPPS)(L)(L’)] complexes present in the solutions bond to the Fe(II) in the next molecule via an O atom of one of the -SO_3_^−^ so that the porphyrins form at least one staggered stack. We would expect a similar bonding in the [Fe(II)(TNPS)(L)(L’)] complexes but the stack may be more disrupted by the bulkier naphthyl groups. Looking at Fig. 3 for a p.q.s. value of -0.49 mm s^− 1^ would generate a *d* parameter of around 21 (in 10^3^ cm^− 1^). This *d* value is we believe reasonable for a *p*-C_6_H_4_SO_3_^−^ that is a substituent group on a porphyrin by comparison to a pure -C_6_H_4_SO_3_^−^ and other values given by Shimura [32] and it would help explain why this axial ligand forms six coordinate low spin [Fe(II)(Por)L_2_] complexes.

In the [Fe(II)(SPh)_2_(SMePh)_2_P)(L)(L’)] system in aqueous solution the low spin complex has a ΔE_Q_ of around 1.41 mm s^− 1^. This can be explained by the presence of one axial ligand being the O atom of the -SO_3_^−^ of a second complex, and the other axial ligand being a H_2_O molecule. Presumably the two (SMep) are not in optimum positions for their -SO_3_^−^ to bond to the Fe(II) atoms. So, in this case complexes contain partial stacks of [Fe(II)((SPh)_2_(SMePh)_2_P)(-C_6_H_4_SO_3_^−^)(H_2_O)] molecules (where the stack could possibly be as small as two of these molecules cross bonding).

One sample of a more concentrated [Fe(II)(SPh)_2_(SMePh)_2_P)(L)(L’)] solution at pH 11.5 manifested a ΔE_Q_ value of 1.21 mm s^− 1^ which was similar to those found for the [Fe(II)(TPPS)(-C_6_H_4_SO_3_^−^)_2_] and [Fe(II)(TNPS)(-C_6_H_4_SO_3_^−^)_2_] frozen solutions and is evidence for both axial ligands being -SO_3_^−^ groups (from neighbouring molecules.) in the concentrated solutions.

We did not find evidence for any low spin [Fe(II)(PPIX)L_2_] complex in frozen solutions over the pH range 7–14 [34, 35], unlike the cases for other [Fe(II)(Por)L_2_] complexes in frozen solution (where Por = TPPS, TNPS, (SPh)_2_(SMePh)_2_P, or T4MepyP). This again shows that PPIX is very different from the man-made porphyrins. In fact, in the latter solutions, we saw evidence for an aggregated intermediate spin Fe(II) complex and two high spin 5 coordinate complexes [34, 35]. We believe the high spin complexes in the Fe(PPIX) frozen solutions and those of the other porphyrins contained at least one OH- axial ligand. Moreover, the low value of the p.q.s. of OH^−^ even in the presence of a water molecule as a possible sixth ligand was not able to stabilize a low spin 6 coordinate complex. Whereas a stronger axial ligand such as CO can stabilise a six-co-ordinate complex as discussed earlier. It is noteworthy that the largest ΔE_Q_ value for an [Fe(II)(PPIX)L_2_] complex is where L=pipyridine (1.42 mm s^− 1^), this is not as large as those found for other [Fe(II)(Por)L_2_] complexes and again shows how PPIX is different [1]. Unlike the other Fe(II)porphyrins (charged porphyrins either anions or cations) discussed above, in aqueous solution Fe(II)(PPIX) does not form an [Fe(II)(PPIX)(H_2_O)_2_] complex instead forming an intermediate spin complex which on increasing the pH reacts with OH^−^ to form high spin complexes [34, 35]. The two neutral iron porphyrins [Fe(II)(TPP)] [10] and [Fe(II(OEP)] [38] are both intermediate-spin (S = 1) complexes with very similar Mössbauer parameters to the intermediate-spin [Fe(II)(PPIX)] complex we previously presented [34, 35]. Further evidence for this assignment is found in the δ values of these intermediate-spin complexes [10, 34, 35, 38] which are larger than those of the low spin complexes discussed in this work.

**Fig. 4:**
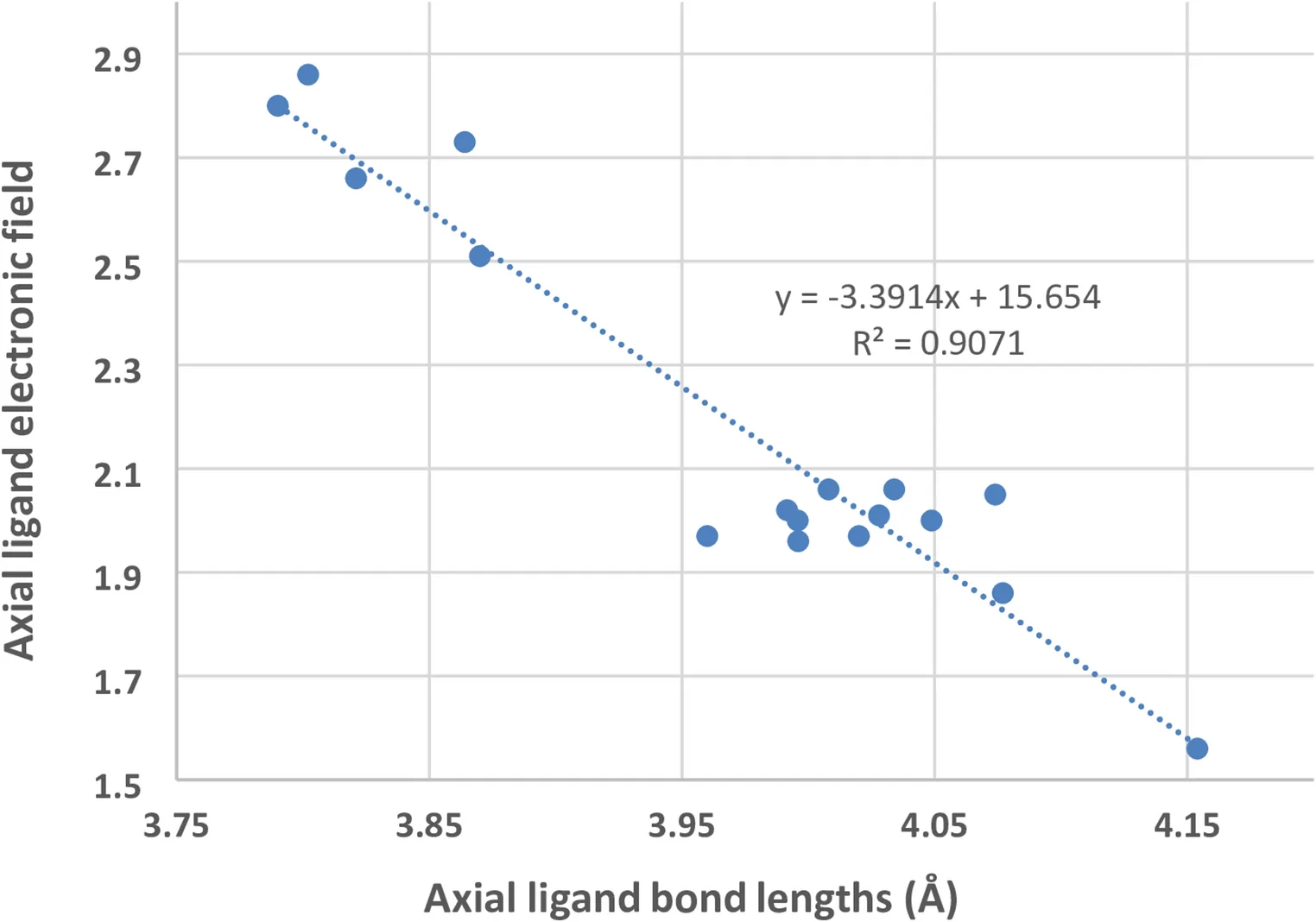
Plot of the electronic field generated by the ligands (equivalent to 4(p.q.s.) _B_ of Eq. 1 in the text above) against the sum of the two axial bond lengths for the complexes where the structures are tabulated in Table 2 (for the Fe(II)(Por) complexes where Por = TPP (nine complexes), Tpiv (four complexes), OEP (one complex), TMP(two complexes) and PMXPP (one complex)

In Fig. 4a plot of the relationship between the axial ligand field as defined in Eq. 1 against the sum of the two axial bond lengths for the complexes in Table 2. In this plot to calculate the values of 4(p.q.s.)_B_ (see Eq. 1 adapted below)

$$ {\mathrm{4}}\left( {{\mathrm{p}}.{\mathrm{q}}.{\mathrm{s}}.} \right)_{{\mathrm{B}}} = {\text{ 4}}\left( {{\mathrm{p}}.{\mathrm{q}}.{\mathrm{s}}.} \right)_{{\mathrm{A}}} - \Delta E_{Q} $$

For 4(p.q.s.) _A_ we used the p.q.s. values for the porphyrins we previously derived in ref 1 tabulated herein in Table 3 and deducted the *ΔE*_*Q*_ value for the complex. We missed this relationship in our previous work [1] but have realised its importance here as it validifies the importance of the point charge model in finding p.q.s. values for ligands around the Fe(II) atom in the complexes. Clearly the R^2^ value for the trend line in Fig. 4 is very reasonable. We omitted two TMP complexes from Table 2 as we discussed these previously [1] in these the haem plane is ruffled so the D_4h_ symmetry brakes down, we also omitted the Por complexes where the p.q.s. value of the porphyrin was not established due to a limited number of complexes. For the Ppiv_2_ C_12_P we used the p.q.s. value for Tpiv. From the plot it appears that the point representing [Fe(II)(TPP)(CCl_2_)(H_2_O)] is the furthest from the trend line. This complex has the shortest Fe-N_Por_ distance (1.984(4) Å) and as this could be significant, we decided to increase the number of complexes we had in the plot by adding the data for the complexes listed in Table 5. The main result displayed in Fig. 4 is that the larger axial field is related to the smallest axial ligand bond lengths.

**Table 5:** Crystal Structure data for low spin [Fe(II)(Por)(NO)_2_L] complexes and [Fe(II)(Por)(NO)L] complexes

Compound	Fe-CO, *N*(NO_2_)-Fe, Fe-*N*(NO)- or Fe-CN Å	*N*_Por_-Fe Å	Fe-L_Ax_ Å	Δ^*^	ΔE_Q_ (mm s^− 1^)	Refs.
[Fe(II)(OEP)(CO)_2_]	1.8558(10)	2.0113(7)		0.0	0.18	39
[Fe(II)(OEP)(py)(CS)]	1.707(2)	2.005(2)	2.1469(18)		0.67	40
[Fe(II)(OEP)(1-MeIm)(CS)]	1.703(4)	2.001(4)	2.112(3)		0.47	40
[Fe(II)(TpivPP)(NO_2_)(CO)]^−^ molecule(1)	2.006(4)	1.992(7)	1.782(4)	0.08^a^	0.32	41
[Fe(II)(TpivPP)(NO_2_)(CO)]^−^ molecule(2)	2.009(4)	1.997(6)	1.789(5)	0.05^a^	0.32	42
[Fe(II)(TpivPP)(NO_2_)(PMS)]^−^	1.937(3)	1.990(6)	2.380(2)	0.04^b^	1.18	43
[Fe(II)(TpivPP)(NO_2_)(py)]^−^	1.951(5)	1.990(15)	2.032(5)	0.01^c^	0.93	43
[Fe(II)(TpivPP)(NO_2_)(NO)]^−d^	2.086(8)	1.988(6)	1.792(8)	0.10^e^	1.78	44
[Fe(II)(TpivPP)(NO_2_)(NO)]^−d^	2.080(8)	1.992(6)	1.774(8)	0.08^e^	1.78	44
[Fe(II)(TpivPP)(NO_2_)(NO)]^−f^	2.060(7)	1.986(6)	1.840(6)	0.09^e^	1.20	44
[Fe(II)(TPP)(1-MeIm)(NO)]^g^	1.743(4)	2.008(12)	2.180(4)	0.07^h^		45, 46
[Fe(II)(TPP)(1-MeIm)(NO)]^i^	1.750(2)	2.008(13)	2.173(2)	0.04^h^	0.73	45
[Fe(II)(TPP)(4-MePip)(NO)]^g^	1.721(10)	2.004(9)	2.328(10)	0.09^j^		45, 46
[Fe(II)(TPP)(4-MePip)(NO)]^i^	1.7517(19)	2.008(8)	2.2851(19)	0.06^i^	0.91	45
[K(222)]_2_[Fe(II)(TPP)(CN)_2_]	1.969(4)	1.990(4)		0.01	0.24	47
[K(222)]_2_[Fe(II)(TPP)(CN)_2_]	1.976(4)	1.999(6)		0.09	0.13	47
[K(222)] [Fe(II)(TPP)(1-MeIm)(CN)]	1.929(3)	1.988(3)	2.048(2)	0.03	0.61	47

**Fig. 5:**
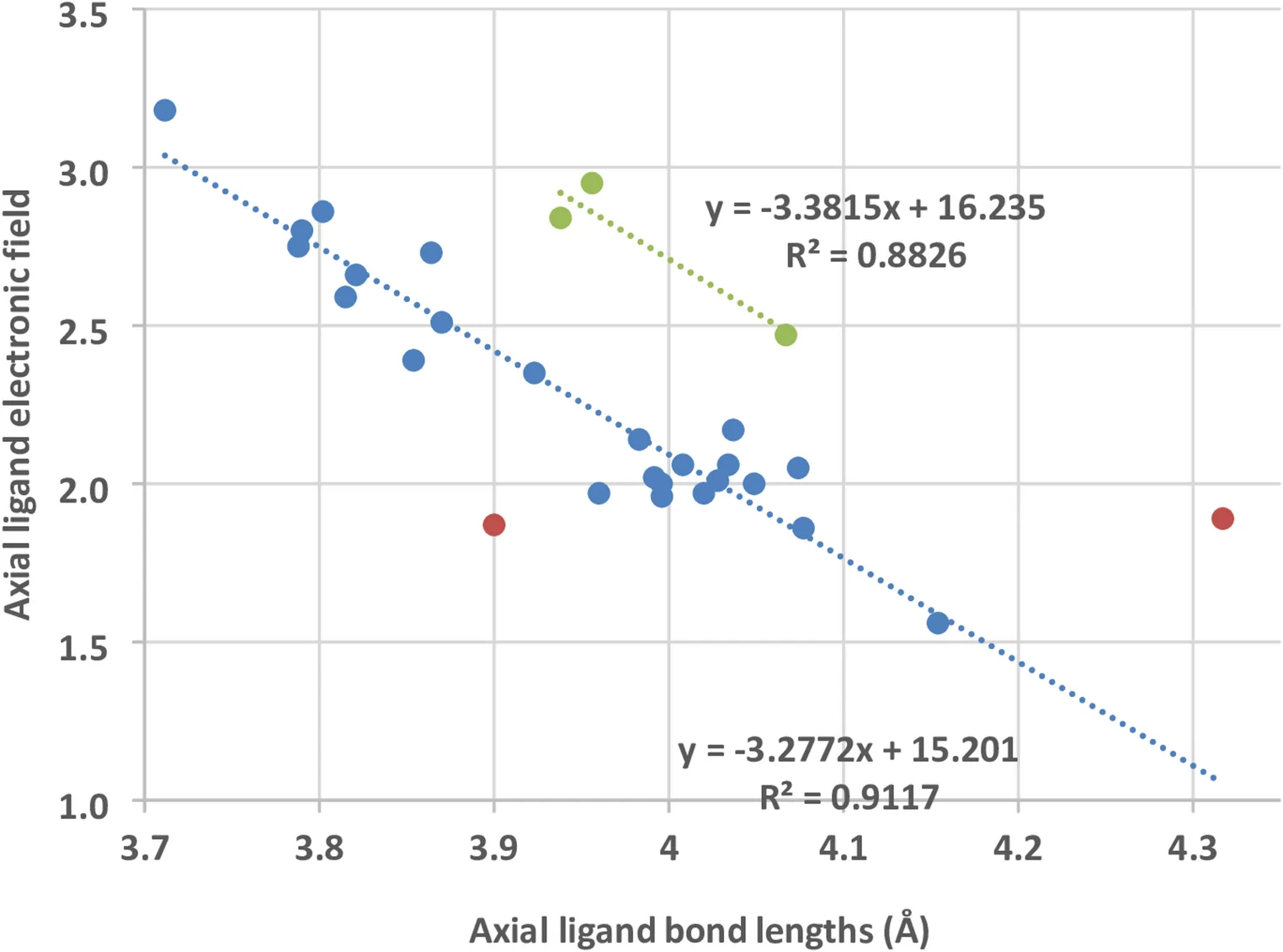
Plot of the electronic field generated by the ligands (equivalent to 4(p.q.s.)_B_ of Eq. 1 in the text above) against the sum of the two axial bond lengths for the complexes given in table two and displayed on Fig. 4 as well as most of the complexes from Table 5 as explained in the text

Displayed in Fig. 5 are the 17 complexes used in Fig. 4 with an additional 12 complexes (see Table 5) of these three are Fe(II)OEP complexes [39, 40], four are Fe(II)(Tpiv) complexes containing an with an NO or NO_2_ axial ligands or both [41–44], two are Fe(II)(TPP) [45–46] containing an NO ligand and three TPP complexes containing CN^−^ axial ligands [39] (Table 6). Of these seven complexes fit onto the trend line shown in Fig. 4 taking the number on the trend line to 24 complexes. The two complexes shown as orange spots are for the complexes[Fe(II)(Tpiv)(NO_2_)(PMS)] [43] and [Fe(II)(Tpiv)(NO_2_)(NO)] [44] ; the former has the shortest Fe(II)-N_Pr_ bonds as well as the longest Fe(II) to axial ligand (PMS) bond, whereas the latter has the largest Fe(II) displacement from the porphyrin plane. The three complexes containing cyanide [47] are all on the upper side of the main trend line and this is quite surprising and shows they behave differently to the other [Fe(II)(Por)(L)(L’)] complexes. Firstly, we were previously unaware [1] of this profound difference and derived p.q.s. values of -0.73 mm s^− 1^ from the [Fe(II)(TPP)(CN)_2_] complex, -0.76 mm s^− 1^ from the [Fe(II)(PPIX)(CN)_2_] complex and − 0.77 mm s^− 1^ from the [Fe(II)(TPPS)(CN)_2_] complex using Eq. 1, (see Tables 1 and 7), and though these values still seem reasonable, clearly something else is important in these complexes as indicated in Fig. 5. For these cyanide complexes to fit on the trend line there combined axial bond lengths would have to be shorter than those observed in the crystal structures. Li et al. [47] suggest that these CN^−^ complexes have low lying-lying excited states because of the large temperature dependence of the *ΔE*_*Q*_ values of the two bis cyano derivatives [47] but this cannot be the entire explanation as the *ΔE*_*Q*_ of [Fe(II)(TPP)(1-MeIm)(CN)]^−^ [47] does not have a large temperature dependence. It is worth noting that Bancroft [29] and Nemykin [31] used a value of -0.84 mm s-1 for CN^−^ which is larger than the values that we derived [1]. However, if we used their value then the [Fe(II)(TPP)(CN)_2_] complexes would be much further from the trend line in Fig. 5. This is probably evidencing that CN^−^ interacts with the [Fe(II)(TPP)] moiety in a more complex way, this may be due to the fact that this ligand carries a negative charge. The location of this electron in the molecules has not been established, it may delocalise onto the porphyrin causing the latter to have very different properties in the way it bonds to the Fe(II) atom, or it may remain entirely on the CN^−^ ligand. We note in the case of [Fe(II)(Pc)(CN)_2_] the Pc ligand is a much better σ- donor than the Por ligands. So, an explanation is that in these cyanide complexes the TPP has a different p.q.s. value compared to its value in the other TPP complexes studied in this work and previously [1]. It is more likely that the negative charges on the CN^−^ axial ligand cause a significant *q*_*Latt*_ contribution to the *ΔE*_*Q*_ (greater than 10%). This is important because such an effect cannot be ignored in calculating p.q.s. values (see discussion in ref 1) and compromises the point charge model. We also note that Li et al. [47] comment on the stretching frequencies of the CN^−^ complexes and the differences compared to the analogous CO complexes, They found that in contrast to the CO complexes the differences in the CN^−^ stretching frequencies in the CN^−^ complexes was very small. We previously discussed the stretching frequencies of a wide range of [Fe(II)(Por)(L)(CO)] and Fe(II)(Por)(CO)_2_] complexes (see Fig. 5 of ref 1) and agree with Li et al. [47] that the behaviour of the CN^−^ complexes is surprisingly. This we believe may also suggest that the charge on the cyanide ligands may be affecting the properties of the complexes. It is worth noting that typical Fe(II)-CN bond lengths in inorganic salts of the type Na_3_[Fe(II)(CN)_5_(NO)].H_2_O (similar to the salts used by Bancroft et al. [29] to derive their p.q.s. value for CN^−^) range between 1.90 Å and 1.92 Å [48]. These values are smaller than those of Li et al. [47], moreover, the stretching frequency for the Fe(II)-CN^−^ bond in K_4_[Fe(II)(CN)_6_] is 2044 cm^− 1^ [49] which is lower than those of Li et al. [47]. Finally in a classic paper by Caughey et al. on CN^−^ as an infrared probe of heamprotein ligand binding sites a stretching frequency of 2024 cm^− 1^ is given for reduced horse-peroxidase CN^−^ (horse-radish) [50] In all these cases the stretching frequencies are lower than those reported by Li et al. [47], and all would be in keeping with rigid Fe(II) electronic environments caused by “lattice charge” affects.

**Table 6:** Ligand p.q.s. values from Tables 6 and 7 of ref 1 and *d* (10^3^ cm^− 1^) values from ref 32

Ligand	*p*.q.s. mm s^− 1^	d(10^3^ cm^− 1^)	Comments
CO	-0.80	35.0	
CN^−^	-0.75	32.1	Average of our value given herein.
P(OMe)_3_	-0.65	29.4	
PMe_3_	-0.655	25.6	
NO_2_	-0.57	25.3	
PPh_3_	-0.55	23.2	p.q.s. value taken from PBu_3_ (see ref. 1)
(CH_2_NH_2_)_2_	-0.472	21.4	
Im	-0.52	21.2	
NH_3_	-0.52	21.0	
Py	-0.46	20.8	
MeNH_2_	-0.49	20.0	p.q.s. value taken from PrNH_2_ (see ref 1)
H_2_O	-0.3225	16.5	p.q.s. value calculated in this work
OH^−^	-0.24	15.4	p.q.s. value calculated in this work

A final point on the p.q.s. values we have derived for CN^−^ (of between −0.73 mm s^− 1^ and − 0.77 mm s^− 1^) for the [Fe(II)(TPP)(CN)_2_] complexes in Table 7 being reasonable is seen from the position of these on the plot in Fig. 3 (see Table 6 for the data). A larger value for the p.q.s. of CN^−^ such as that of Bancroft [29] (-0.84 mm s^− 1^) would have shown up much further from the trend line given in Fig. 3. However, based on the discussion above it is likely that the true p.q.s. value for CN^−^ is somewhere between those of ours and that of Bancroft et al. [29]. For clarity we have added Table 7 below, which is adapted from Table 10 of ref [1] but also contains infra-red data taken from ref 47.

**Table 7:** ^57^Fe Mossbauer data for low spin and CN stretching frequencies (ν) (where known) for [Fe(TPP)(CN)_2_]^2−^ and related complexes.;

Compound	T K	δ_obs_ mm s^− 1^	δ_calc_ mm s^− 1^	Δ_obs_ mm s^− 1^	Δ_calc_ mm s^− 1^	ν CN^−^ cm^− 1^	Ref.
[K(222)] [Fe(TPP)(CN)(1-MeIm)]	298 100 16	0.35^a^ 0.46 0.48	0.34	0.62^**b**^ 0.61 0.60	0.62	2076	47
[K(222)]_2_[Fe(TPP)(CN)_2_]	298 100 20	0.31 0.40 0.40	0.31	0.13 0.24 0.27	+ 0.18	2069	47
[K(222)]_2_[Fe(TPP)(CN)_2_]	298 100 15	0.31 0.38 0.38	0.31	0.07 0.13 0.15	+ 0.18	2056	47
[Fe(II)(PPIX)(CN)_2_]^2−^	77	0.43	0.33	0.0	0.0		1
[Fe(II)(TPPS)(CN)_2_]^2−^	77	0.33	0.34	0.0	0.0		1

^a^ Used to calculate p.c.s. value for CN^−^; ^b^ used to calculate p.q.s. value for CN^−^; ^c^ infra-red data taken from ref47

### p.c.s. values

We now turn to the p.c.s. values for these ligands. The small p.c.s. and p.q.s values found for H_2_O and OH^−^ plotted on Fig. 11 of ref 1 are interesting, whereas those for the former are just off the red line towards the top, those of the latter are just above the area of the plot. Thus, the plot illustrates how these very weak ligands differ from the ligands that easily form stable compounds with [Fe(II)(Por)L_2_] complexes.

In addition, only very strong π- bonding ligand such as CO can stabilise OH^−^ in a low spin 6 coord [Fe(II)(Por)(CO)(OH)] complex. It is interesting that the p.q.s. value for OH^−^ is 0.01 mm s^− 1^, it indicates that this ligand puts very little electron density onto the Fe(II) atom.

The p.c.s. values for the porphyrins change for complexes containing the pairs of axial ligands CO + H_2_O or CO + OH^−^. The values are less than for two non-CO ligands but not as low as CO + N-type ligand.

The p.c.s. value for the carbenium ion CCl_2_ is significantly large and is the most positive of any axial ligand we have seen to date showing that it is pushing electron density onto the Fe(II) atom when it bonds.

The p.c.s. value for t-butNC (0.02 mm s^− 1^) is small like that of CN^−^ which is expected, the value is similar to that derived from the [Fe(II)(Pc)L_2_] complexes [31] if 0.06 mm s^− 1^ is subtracted. We note that all the p.c.s. values of ref [31] are consistently 0.06 mm s^− 1^ more positive than ours, and we believe the reason for this is there δ values were given relative to sodium nitroprusside and calculated from room temperature data. It is also worthy of note that the p.q.s. value of -0.72 mm s^− 1^ [31] is also close to our value of -0.715 mm s^− 1^.

The -SO_3_^−^ groups on TNPS and TPPS have similar p.c, s, values to those of the aliphatic amines we discussed previously [1] showing they bond strongly to the Fe(II) atom, and there p.q.s. values are also similar to those of the aliphatic amines. It is therefore not surprising that they can act as axial ligands tin [Fe(II)(Por)L_2_] complexes in aqueous solutions.

### Relevance to haemoglobin and myoglobin and other haem proteins

From the p.q.s. values derived in this paper and in ref 1, we can calculate that the [Fe(II)(PPIX)(Im)(H_2_O)] entity would have a *ΔE*_*Q*_ value of 1.395 mm s^− 1^ which would be a low spin complex. We have found no evidence of such a species in all our studies on [Fe(II)(PPIX)(L)_2_] (where L is an aliphatic aromatic or other heterocyclic nitrogen binding ligand) complexes [51–53]. In fact, we provided evidence for the complexes all binding two nitrogenous ligands by preference [51, 52]. However Fe(PPIX) (also known as haem b) is the prosthetic haem found in many haem proteins, either with one axial ligand such as a histidine residue (as in haemoglobin or myoglobin) or two axial ligand histidine residues (as in the b-type cytochromes) or with a methionine as an axial ligand (as in cytochrome P450) also bonded to the protein leaving the other axial site open for other molecules to bind (depending on the role of the protein). It has been suggested that in deoxyhaemoglobin) (and deoxy myoglobin) a water molecule could replace the oxygen molecule, this cannot be the case as the entity would (from this calculation) be low spin Fe(II) and not as observed high spin Fe(II). To eliminate the possibility of a water molecule binding the access to the open axial site has therefore to be hydrophobic as water is abundant in living cells.

Before providing further evidence to back the above explanation we need to refer to discussion on some weak-binding S-type ligands that we have previously reported [1] and derived p.c.s. and p.q.s. values for. The complexes we used to derive the values were originally used to model cytochrome P450_Cam_ [54–56]. We discussed the fact that in frozen solution and in the solid state all the S ligands studied by Mössbauer spectroscopy to date all formed five coordinate high spin [Fe(II)(PPIX)L] (where L = a sulfur bonding ligand) complexes, and these could only be converted to low spin six coordinate[Fe(II)(PPIX)(CO)L] complexes by adding CO to the solutions before freezing [1]. These six coordinate complexes all manifested δ values in the range 0.30 to 0.37 mm s^− 1^ at 78 K and *ΔE*_*Q*_ values in the range 0.6 to 0.95 mm s^− 1^. The *ΔE*_*Q*_ values yielded p.q.s. values in the range − 0.335 to -0.23 mm s^− 1^ [54, 55] except for that of ethyl 2-mercaptoacetate which has a calculated p.q.s. of -0.40 mm s^− 1^ which is high. (we note without the CO ligand only the high spin five coordinate [Fe(II)(PPIX)] complex forms). We also noted that the two [Fe(II)(TPPS)(CO)L] complexes had calculated p.q.s. values that were also high (-0.465 mm s^− 1^ and − 0.475 mm s^− 1^) and are different from those of the equivalent [Fe(II)(PPIX)(CO)X] complexes. It was clear that something is happening in the bonding on the [Fe(TPPS)(CO)L] complexes. Thus, for the four complexes of the type [Fe(II)(Por)(CO)L] [54–56] which apparently have p.q.s. values that are more like nitrogenous ligands the environment around the S-bonding ligand is affecting the contribution the ligand is making to the electric field across the Fe(II) atom. We noted that all four complexes have typical hyperporphyrin spectra with the Soret band between 336.5 nm and 451 nm. However, in all cases the four ligands do not form low spin [Fe(Por)L_2_] complexes [54–56].

For the four cases where the p.q.s. values were between − 0.335 and − 0.23 mm s^− 1^ low spin complexes of the type [Fe(II)(PPIX)L_2_] also do not form [54, 55]. We pointed out that if they did, then if we take the value range of -0.335 to -0.23 mm s^− 1^ with [Fe(II)(PPIX)] we would expect *ΔE*_*Q*_ values in the range ~ 1.70 to 2.12 mm s^− 1^. Such values were larger than anything yet found for low spin [Fe(II)(PPIX)L_2_] complexes, hence we ruled out the possibility of finding six co-ordinate low spin [Fe(II)(PPIX)L_2_] complexes with such values.

This as we previously stated [1] is a very significant finding as it sets a limit to the *ΔE*_*Q*_ value range for low spin complexes of the type [Fe(II)(PPIX)L_2_]. Thus, these sulfur ligands cannot form low spin [Fe(II)(PPIX)LL’] complexes, but when CO is present as the other axial ligand, they can form [Fe(II)(PPIX)(CO)L] complexes [54–56].

Interestingly, in the c-type cytochromes six-coordinate-low-spin Fe(II) haem complexes form the prosthetic groups. In these cytochromes the vinyl groups of a haem b have been involved in forming thioether bonds with cystine residues donated by the protein, thereby covalently linking the haem to the protein [57], the resulting entity is often referred to as haem c. The Fe(II) atom in the resulting modified “[Fe(PPIX)]” is said to have a different affinity for binding ligands (compared to unmodified [Fe(II)(PPIX)] and the binding constant of the ferrous form for sulfur ligands is enhanced [57], and so in cytochromes c the haem bs bind their methionine ligands very tightly [57]. The surprisingly high p.q.s. values for some of the S-ligands we found from four of the complexes above (where we commented that other effects must be tightening the bonding of the S-ligands may be relevant to the bonding in c-type-Fe(II) haems [1]. In all the c-type cytochromes histidine (N) and methionine (S) are present as the axial ligands bonding to the Fe(II) in the haem [57]. The Mössbauer parameters of several c-type cytochromes have been reported [58–60]. The first reported study was carried out on the reduced and oxidised forms of horse cytochrome c [58]. In another case:- Cytochrome c from Methilococcus capsulatus was enriched with ^57^Fe in vivo [60] and was studied to understand the dynamics of the protein; it was found that upon reduction the flexibility of the protein was diminished. It was suggested that the differences in flexibility were sufficient to explain the differences in physiochemical properties between the oxidised and reduced forms [60].

As we explained if the p.q.s. values for histidine and ethyl 2-mercaptoacetate, (EMCA), derived in our previous work [1] were used with that for [Fe(II)(PPIX)] to calculate a *ΔE*_*Q*_ value for [Fe(II)(PPIX)(His)(EMCA)] the result is 1.24 mm s^− 1^. This *ΔE*_*Q*_ value is close to those reported for the cytochromes c and shows the benefit/validity of our approach [1]. Differences from the calculated value of 1.24 mm s^− 1^ may also indicate that the covalent linking to the protein effects the environment around the haem and thus modifies the electronic properties/bonding of the N ligands of the porphyrin which in turn effects their bonding to the Fe(II) atom in addition to affecting its position during the oxidation/reduction cycle. Relevant to this argument it has been reported that “a nonplanar distortion of the haem of c-type cytochromes is conserved in the proteins isolated from diverse species based upon a comprehensive analysis of available high resolution X-ray crystal structures” [61]. This was true for both the oxidised and reduced forms [61]. Indeed, it was reported that this distortion was induced through the cysteine thioether linkages between the porphyrin pyrrole groups and the polypeptide and results in an asymmetric pyrrole distortion [61]. This distortion is manifested as a ruffling of the porphyrin and is also conserved [61]. The authors also point out that for other haem proteins which lack such covalent bonds to the porphyrin moieties, nearly planar porphyrins are observed [61]. It is highly likely in our opinion that it is the thioether linkages which causes a modification of the p.q.s. value of the haem c N ligands relative to those of PPIX.

Previously we noted [1] that if the p.q.s. value for thiophenol (which is not similar to methionine) had been used with that of histidine, the resulting *ΔE*_*Q*calc_ value would have been 1.58 mm s^− 1^ which as discussed is towards the extreme end of stable low spin Fe(II) complexes. As the p.q.s. value for histidine is amongst the largest of the nitrogenous ligands then other such ligands with smaller p.q.s. values would not be able to form six co-ordinated complexes with [Fe(II)(PPIX)] and a S bonding ligand. This emphasizes the important choice that natural processes have arrived at with evolving histidine as the fifth ligand for [Fe(II)(PPIX)]. Clearly histidine residues have the advantages of size, shape, available electron density and orbitals for bonding, which together must satisfy the requirements of the haem proteins. Presumably, the kinetic and thermodynamic properties of the histidine residues are the main principles/drives in the evolution of these important molecules for oxygen transport and storage.

Further evidence for our explanation can be found in the relatively new field nonnatural haem proteins. These haem proteins have been engineered by directed evolution to catalyse chemical transformations that to our knowledge are biochemically unprecedented. One such enzyme is derived from thermostable *Rhodothermus marinus* (*Rma*) cytochrome c [61]. A high-resolution crystal structure has shown that this enzyme has a haem c with a histidine ligand from the protein as its fifth ligand and either a water molecule or an OH^−^ as the other axial ligand [61]. The haem c is also connected to the protein via two cysteine links as it is in cytochrome c, so although it is related to to Fe(PPIX) it is not the same and we would have expected it to have slightly different p.c.s. and p.q.s. values. In addition, the authors have shown that the haem plane is ruffled. The protein has a *ΔE*_*Q*_ *=* 1.18 mm s^− 1^ and a *δ =* 0.54 mm s^− 1^. Although this is less than the *ΔE*_*Q*_ of 1.395 mm s^− 1^ (we calculated using the p.q.s. value we derived for PPIX it is in the range we might expect if haem c is different from Fe(II)(PPIX), which it clearly is. We note that the authors point out that this is a low spin Fe(II) complex unlike those in other proteins (Fe(II)(PPIX)) lacking an endogenous distal ligand to the Fe (the later haem tends to be flat) which have been shown to prefer five-coordinate high spin states, when oxygen is not bound [61]. They also point out that when a carbene replaces the OH^−^/water (in carbene bound *Rma* TDE) the haem plane is still very ruffled and the resulting *ΔE*_*Q*_ = 1.83 mm s^− 1^. This as they note is a very large value. We found it cannot be used to derive a p.q.s. value as large as those we found for CCl_2_ and t-butNC, in fact a derived p.q.s. value would be very small. We believe that ruffling and strained/distorted structure around the Fe atom are major factors that affect the Fe(II) *ΔE*_*Q*_, in these haem c proteins. It should be noted that the theory we are using to calculate the p.q.s. values depends on the symmetry of the Fe atom being *D*_*4h*_. Indeed, only small asymmetry values (η values) are in keeping with *D*_4h_ symmetry at the iron site, clearly the distortions we have discussed are affecting the symmetry as witnessed by not being amenable to giving reasonable p.q.s. values for the carbene ligands. We further note that the large *ΔE*_*Q*_ values found for [Fe(II)(TFPP)(CPh_2_)(1,2-Me_2_Im)] (*ΔE*_*Q*_ − 1.82 mm s^− 1^) and [Fe(II)(TFPP)(CPh_2_)(1-EtIm)] (*ΔE*_*Q*_ = 1.91 mm s^− 1^) are also associated with highly ruffled porphyrin planes [62]. In addition, the CPh_2_ ligands form strong and “hard” axial carbene bonds (Fe-C) but “flexible” trans ligand bonds (Fe-N_Im_) which are longer than those of most other [Fe(II)(Por)L_2_] (L = Im type ligand) complexes [62] and also show larger tilting angles (that tilt off the z-axis). Clearly though [Fe(II)(TFPP)] can be used to model some properties of haem c it is clearly very different to other porphyrins and its steric hindrance caused by its four -C_6_F_5_ substituent groups makes it behave very like [Fe(II)(TMP)] which we have discussed at length [1]. The finding that the sequence of Fe-N_Por_ distances is consistent with that of the porphyrin plane conformations, for which more ruffled porphyrin planes correspond to shorter (Fe- N_Por_) distances [62] we find interesting, as we reported the Mössbauer parameters and crystal structure of [Fe(II)(TFPP)(Fe(C_5_H_5_)(C_4_H_4_N))_2_] [19, 63]. This complex manifested little ruffling and had long Fe-N_Por_ distances of 2.01(2) Å [19], which are at the longer end of those reported in ref 63, and its *ΔE*_*Q*_ is 1.25 mm s^− 1^ [19] which is much smaller and consistent with a less distorted electronic environment around the Fe(II) atom, in addition the Fe-N axial bond lengths to the azaferrocene moieties are also long (2.05(2) Å) [63]. Whereas the bulk of the azaferrocene ligands might have been expected to distort the structure. As we had azaferrocene ligands present the Mössbauer data for these moieties gave us a ligands eye view of the bonding to the Fe in the TFPP plane; we were able to reason that this was through σ bonding from the lone pairs of electrons on N atoms of the azaferrocenes with little or no π-back-bonding from the Fe in the TFPP plane [19]. Our Mössbauer data were verified by other workers [64]. Thus, from this discussion when ruffling of the haem plane takes place the symmetry around the Fe(II) atom distorts away from D_4h_ symmetry and loses the D_4_ axis and the theory no longer works.

We previously drew attention to the fact that the *ΔE*_*Q*_ values for both oxyhaemoglobin and oxymyoglobin were too large to be those of low spin Fe(II) complexes [1] and from this work it is apparent that oxygen bonding ligands such as water and OH^=^ do not form stable low spin [Fe(II)(PPIX)(L)(L’)] complexes (where L is a nitrogenous ligand and L’ is H_2_O, OH^−^ or O_2_). A recent overview of the state of the art of haemoglobin chemistry [65] suggests that the nature of its bonding to the oxygen molecule is still not fully understood. We believe that our findings offer more support for the Weiss model [66, 67] (of Fe(III) bound to a superoxide molecule) and we will offer further support for this in a coming paper.

## Conclusions

These findings again illustrate how the use of p.q.s. values can aid in our understanding of [Fe(II)(Por)L_2_] and [Fe(II)(Por)(L)(L’)] complexes and related haem proteins.

From the p.q.s. value derived herein for H_2_O we were able to calculate the theoretical *ΔE*_*Q*_ value for [Fe(II)(PPIX)(His)(H_2_O)] as -3.04 --2x(his(p.q.s)--2 × 0.3225 = -3.04–1.00–0.645 = 1.395 mm s^− 1^ and compare it to some haem proteins such as deoxyhaemoglobin and deoxy myoglobin showing these complexes would be low spin Fe(II) if they contained a water ligand. The fact that they are not low spin Fe(II) proves the absence of a water molecule.

In contrast we were able to show that haem c in the cytochromes c was able to form a six-coordinate low-spin with a water molecule or OH^−^ as the sixth axial ligand and that this was because its porphyrin plane was ruffled by forces exerted via the cysteine links from the protein.

In this work and in our previous studies [1] we have shown that Fe(II)(PPIX) has unique properties that synthetic porphyrins (such as TPP) cannot adequately mimic. This is because the synthetic models have periphery groups on the porphyrins that alter both the way the model compounds interact with other molecules in solution, and how their periphery substituents modify the bonding properties of the four inner nitrogen ligand atoms. The latter modifications to the four nitrogen chelating atoms are reflected in the different p.c.s. and p.q.s. values we have derived here and previously for the man-made porphyrins [1].

## Experimental

[Fe(II)(TPP)(CCl_2_)(H_2_O)] was prepared as described in the literature [8] and its electronic absorption spectrum manifested close agreement (see Table 4). The crystal structure [8] (see Table 2) showed the octahedral coordination of the low spin [Fe(II)(TPP)(CCl_2_)(H_2_O)] compound. The Mössbauer data collected herein are presented in Table 1.

[Fe(II)(TPP)(t-butNC)_2_] was also prepared by the literature method [9]. The electronic absorption spectrum presented in Table 4 is very close to the literature data [9]. Crystal structure data are listed in Table 2 [9]. The infra-red spectrum manifested a weak band at 2125 cm^− 1^ corresponding to a CN stretching vibration and compared fairly well to the literature value of 2129 cm^− 1^ (Nujol Mull) [9]. The Mössbauer data are given in Table 1 and the Mössbauer spectrum is shown in figure S1 of the supplementary material.

The electronic absorption spectra for [Fe(II)(TPPS)] and [Fe(II)(TNPS)] solutions have been previously described [5, 6]. Those for [Fe(II)(SPh)_2_(SMePh)_2_P)] and [Fe(II)(T_4_MepyP)] were recorded in the same way on the same instrumentation. All the electronic absorption spectra are presented in Table 4.

Preparation of the frozen solutions of [Fe(II)(TPPS)] and [Fe(II)(TNPS)] used for Mössbauer spectroscopic experiments have been previously described [5, 6]. For the [Fe(II)(SPh)_2_(SMePh)_2_P)] frozen solutions used for the Mössbauer spectroscopic experiments, 200 mg of [Fe(III)(SPh)_2_(SMePh)_2_P)] were dissolved in 2 ml of water and placed under a nitrogen atmosphere. A few drops of concentrated sodium dithionite was then added to the solution. The pH of the solutions was adjusted as required by adding 1 N HCl for pH < 12 or by adding 2 N sodium hydroxide for pH > 12. Frozen samples were prepared under nitrogen and loaded in a nylon cell. One sample made up to pH 11.5 was on a more concentrated solution of [Fe(III)(SPh)_2_(SMePh)_2_P)] containing 250 mg in 2 ml of water.

In the case of the frozen solutions of [Fe(II)(T_4_MepyP)] used for the Mössbauer spectroscopic experiments, solutions contained between 50 and 150 mg of [Fe(II)(T_4_MepyP)] in 2-3 ml of aqueous solution. The solutions were studied above pH 2.0 and the pHs were adjusted using 1 M HCl as required for low pH values in a nitrogen atmosphere. The solutions samples were transferred to nylon cells and frozen in liquid nitrogen.

The electronic absorption spectra (Table 4) and the Mössbauer spectra (Table 1) of both [Fe(II)(T_4_MepyP)((Im)_2_] and Fe(II)(T4MepyP))(CO)(H_2_O)] were consistent with those of low spin six coordinate complexes (with Mössbauer parameters close to similar complexes we have previously published [1]). The former complex was included to facilitate the calculation of a p.q.s. value for T_4_MepyP.

[Fe(II)(PPIX)(CO)(OH)]^−^ was prepared in aqueous solution at pH 9 in the presence of dithionite (which acts as a reducing agent). The stoichiometry of CO bound [Fe(II)(PPIX)] was established by titrating aqueous solutions of the Fe(II)(PPIX) with a solution of CO gas dissolved in water, (this method has been described elsewhere [68]. The exact concentration of CO in the titrant was determined independently by titration of myoglobin at pH 7 with the knowledge that this haem protein binds one CO molecule per haem (see figure S2 of the supplementary data which presents a CO titration of aqueous Fe(II)(PPIX). The binding stoichiometry is clearly of one CO/[Fe(II)(PPIX)]). This value is expected as previous literature [69] has shown that in coordinating solvents only one CO molecule can bind per [Fe(II)(PPIX)]. The Mössbauer spectrum of a frozen solution containing [Fe(II)(PPIX)] and CO (see Table 1 (and figure S3 of the supplementary data)) shows the presence of low spin Fe(II) only and is quite different from Mössbauer spectra collected from [Fe(II)(PPIX)] solutions in the absence of CO [34, 35]. At pH 9 only one low spin iron(II) electronic environment is observed and the *ΔE*_*Q*_ value of 0.94 mm s^− 1^ is concomitant with the presence of a single CO molecule bound per iron atom (the Mössbauer spectrum is shown in figure S3 of the supplementary material). A much smaller *ΔE*_*Q*_ value would be expected if two strong field ligands (e.g. two CO molecules were bound to the iron [1]). For example, where CO and a nitrogen ligand is present where the *ΔE*_*Q*_ value is nearly half of the size of the observed splitting at pH 9 [1]. The observed *ΔE*_*Q*_ value is in fact what would be expected if the second ligand is a weak field ligand such as a hydroxyl or water molecule. The electronic absorption spectrum of [Fe(II)(PPIX)(CO)(OH)]^−^ is presented in Table 4. ^13^C-NMR studies have been made of the CO adduct of Fe(II)(PPIX) [70] and haem proteins [71, 72]. This useful method permits a single carbon atom in the ligand to be observed and to report its environment. We were able to collect spectra of the free [Fe(II)(PPIX)(^13^CO)(OH)]^−^ complex at pH 9 in dimethylformamide/water solutions. In the presence of imidazole in this solvent there was a single peak at 203.5 ppm, whereas in the absence of this ligand, peaks at 205.4 ppm and 210.7 ppm were observed. In purely aqueous solutions at pH 9, no spectral feature could be assigned to either free ^13^CO or [Fe(II)(PPIX)(^13^CO)(OH)]^−^. However, on addition of 10 µM Mn^2+^ EDTA (a chemical relaxing agent), a small broad feature at about 208 ppm, which we believe may reflect the carbonyl complex appeared (data not shown).

Mössbauer spectra were recorded on concentrated frozen solutions at 78 K. The Mössbauer spectrometer and experimental details have previously been described [73].

## Supplementary Information

Below is the link to the electronic supplementary material.

Supplementary Material 1

## Data Availability

No datasets were generated or analysed during the current study.

## Abbreviations

N-AcetylHis: N-Acetylhistidine
TpivPP: “Picket Fence”porphyrin dianion = tetra(o-pivalamidophenyl)porphyrin dianion
1,2-Me_2_Him: 1,2-dimethylimidazole
18-C-6): 1,4,7,10,13,16-hexaoxacyclooctadecane
BzHIm: 1-benzimidazole
1-EtHIm: 1-ethylimidazole
1-MeIm: 1-methylimidazole
1-VinIm: 1-vinylimidazole
2-MeHIm: 2-methylimidazole
2-MeHIm: 2-methylimidazole
2-Mepy: 2-methylpyridine
3NH_2_PzH: 3-aminopyrazole
3-ClPy: 3-chloropyridine
3-EtPy: 3-ethylpyridine
4-CNPy: 4-cyanopyridine
4-NMe_2_py: 4-dimethylaminopyridine
4-MeHIm: 4-methylimidazole
tfpp: 5,10,15,20-tetrakis(pentafluorophenyl)porphyrin dianion
5-MeHIm: 5-methylimidazole
CuIm: A copper imidozalate complex ligand linking to the Fe(III) via an imidazole N atom
L: A nitrogenous aliphatic, an aromatic base or a heterocyclic ligand
Fe(C_5_H_5_)(C_4_H_4_N): Azaferrocene
q_MO_: Charge donation to the Fe atom from the ligands
Clpy: chloropyridine
cMU: Cis -methylurocanate
V_zz_: Component of the efg perpendicular the the porphyrin plan
q_CF_: Crystal field contribution to q_val_
CN^−^: Cyanide
CNpy: Cyanopyridine
Cyt: Cytochrome
CCp: Cytochrome c peroxidase
Efg: Electric field gradient
EPR: Electron Paramagnetic Resonance
EtIm: Ethylimidazole
q_lattice_: Field gradient due to external charges
h.a.l.s: highly axial low spin
His: Histidine
Him: Imidazole
Fe(PPIX): Iron protoporphyrin IX
K(222): Kryptofix-222 or 222, 4,7, 13,16,21,24-hexaoxo-1,10-diazabicyclo[8,8,8]hexacosane
q_latt_: Lattice term contributing to the efg
TMP: Meso- tetramesitylporphyrin dianion
T2,6Cl_2_PP: Meso-tetra(2,6-dichlorophenyl)porphyrin dianion
MeIm: Methylimidazole
Mepip: Methylpiperidine
Mepy: Methylpyridine
Morph: Morpholine
δ(mm s^− 1^): Mössbauer chemical shift
Mb: Myoglobin
n-ButNH_2_: n- butylamine
but: n-butylamine
n-DecNH_2_: n-decylamine
n-DoDecNH_2_: n-dodecylamine
n-OctNH_2_: n-octylamine
n-PrNH_2_: n-propylamine
Q: Nuclear quadrupole moment
OEP: Octaethylporphyrin dianion
OETPP: Octaethyltetraphenylporphyrin dianion
OMTPP: Octamethyltetraphenylporphyrin dianion
octNH_2_: Octylamine
p.q.s: Partial quadrupole splitting
Ph: Phenyl
Pip: piperidine
Porphyrin: Por
PrNH_2_: propylamin
PPIX: protoporphyrin IX dianion
Py: Pyridine
ΔE_Q_ (mm s^− 1^): Quadrupole splitting
λ: Spin orbit coupling constant
TiPrP: Tetra(i-propyl)porphyrin dianion
(2,6-(OMe)_2_)_4_TPP: Tetrakis(meso-2,6-dimethoxyphenyl)porphyrin dianion
TPC: Tetraphenylchlorin
tMU: trans - methylurocanate
q_val_: Valence term contributing to the efg
1-VinylIm: Vinylimidazole
TPPS: α, β, γ, δ – tetra(p-sulfophenyl)porphin dianion
TPP: α, β, γ, δ – tetraphenylporphin dianion
