# Supplementary material for: Protoporphyrin IX iron(II) revisited. overview ofthe Mössbauer spectroscopic parameters of low spin porphyrin iron(II) complexes Part II
Source: J Biol Inorg Chem. 2026 Sep 5;31(6):451–68. doi: 10.1007/s00775-026-02158-9 (PMC13645893; doi:10.1007/s00775-026-02158-9)
Supplement: Supplementary file 1 [file 775_2026_2158_MOESM1_ESM.docx]

**Protoporphyrin IX iron(II) revisited. Overview of the Mössbauer spectroscopic parameters of low spin porphyrin iron(II) complexes Part II**

**Jack Silver^1^ (**[0000-0001-8669-9673](https://orcid.org/0000-0001-8669-9673)**), Jehad A Taies^2^ and George R. Fern^1^ (**[0000-0002-0016-5038](https://orcid.org/0000-0002-0016-5038)**), Husam M Abu-Soud**

^1^ Department of Chemical Engineering, Wolfson Centre for Sustainable Materials Development and Processing, Brunel University of London, Kingston Lane, Uxbridge, UB8 3PH, UK

^2^ Department of Chemistry, College of Education for Pure Science, University of Anwar, Anwar, Iraq

^3^ Department of Obstetrics and Gynecology, Wayne State University School of Medicine, The C.S. Mott Center for Human Growth and Development, 275 E. Hancock Detroit, MI 48201, USA

^*^To whom correspondence should be addressed, Email: [George.Fern@brunel.ac.uk](mailto:George.Fern@brunel.ac.uk)

ORCID: <http://orcid.org/0000-0002-0016-5038>

Figure S1 Frozen solution Mössbauer spectrum of [Fe(II)(TPP)(t-butNC)_2_]

Figure S2 Carbon monoxide titration of aqueous [Fe(II)(PPIX)], The [Fe(II)(PPIX)] concentration was 31 M. The titration was performed at pH 9 (open circles), pH 14 (open squares).

Figure S3 Frozen solution Mössbauer spectrum of aqueous [^57^Fe(II)(PPIX)] in the presence of carbon monoxide at pH 9.
